# Multi-omics approaches reveal the molecular mechanisms underlying the interaction between *Clonorchis sinensis* and mouse liver

**DOI:** 10.3389/fcimb.2023.1286977

**Published:** 2023-11-24

**Authors:** Tingzheng Zhan, Yuhong Wu, Xueling Deng, Qing Li, Yu Chen, Jiahui Lv, Jilong Wang, Shitao Li, Zhanshuai Wu, Dengyu Liu, Zeli Tang

**Affiliations:** ^1^ Department of Parasitology, School of Basic Medical Sciences, Guangxi Medical University, Nanning, China; ^2^ Department of Cell Biology and Genetics, School of Basic Medical Sciences, Guangxi Medical University, Nanning, China; ^3^ Key Laboratory of Longevity and Aging-related Diseases of Chinese Ministry of Education, Guangxi Medical University, Nanning, China; ^4^ Key Laboratory of Basic Research on Regional Diseases (Guangxi Medical University), Education Department of Guangxi Zhuang Autonomous Region, Nanning, China; ^5^ Schistosomiasis Prevention and Control Department, Hengzhou Center for Disease Control and Prevention, Hengzhou, China; ^6^ Department of Immunology, Guangxi University of Chinese Medicine, Nanning, China; ^7^ Guangxi Key Laboratory of Translational Medicine for treating High-Incidence Infectious Diseases with Integrative Medicine, Nanning, China

**Keywords:** *Clonorchis sinensis*, mouse liver, transcriptomics, proteomics, metabolomics, molecular mechanisms

## Abstract

**Introduction:**

Clonorchiasis remains a serious global public health problem, causing various hepatobiliary diseases. However, there is still a lack of overall understanding regarding the molecular events triggered by *Clonorchis sinensis* (*C. sinensis*) in the liver.

**Methods:**

BALB/c mouse models infected with *C. sinensis* for 5, 10, 15, and 20 weeks were constructed. Liver pathology staining and observation were conducted to evaluate histopathology. The levels of biochemical enzymes, blood routine indices, and cytokines in the blood were determined. Furthermore, alterations in the transcriptome, proteome, and metabolome of mouse livers infected for 5 weeks were analyzed using multi-omics techniques.

**Results:**

The results of this study indicated that adult *C. sinensis* can cause hepatosplenomegaly and liver damage, with the most severe symptoms observed at 5 weeks post-infection. However, as the infection persisted, the Th2 immune response increased and symptoms were relieved. Multi-omics analysis of liver infected for 5 weeks identified 191, 402 and 232 differentially expressed genes (DEGs), proteins (DEPs) and metabolites (DEMs), respectively. Both DEGs and DEPs were significantly enriched in liver fibrosis-related pathways such as ECM-receptor interaction and cell adhesion molecules. Key molecules associated with liver fibrosis and inflammation (Cd34, Epcam, S100a6, Fhl2, Itgax, and Retnlg) were up-regulated at both the gene and protein levels. The top three metabolic pathways, namely purine metabolism, arachidonic acid metabolism, and ABC transporters, were associated with liver cirrhosis, fibrosis, and cholestasis, respectively. Furthermore, metabolites that can promote liver inflammation and fibrosis, such as LysoPC(P-16:0/0:0), 20-COOH-leukotriene E4, and 14,15-DiHETrE, were significantly up-regulated.

**Conclusion:**

Our study revealed that the most severe symptoms in mice infected with *C. sinensis* occurred at 5 weeks post-infection. Moreover, multi-omics analysis uncovered predominant molecular events related to fibrosis changes in the liver. This study not only enhances our understanding of clonorchiasis progression but also provides valuable insights into the molecular-level interaction mechanism between *C. sinensis* and its host liver.

## 1 Introduction

Clonorchiasis remains a global foodborne parasitic disease that cannot be ignored. Both humans and mammals can become infected by consuming raw or semi-raw freshwater fish and shrimp that contain *Clonorchis sinensis* (*C. sinensis*) metacercariae (Qian et al., 2016). The adult *C. sinensis* can reside in the human hepatobiliary ducts for a prolonged period, causing inflammation and fibrosis in the vicinity of the bile ducts. Consequently, this parasite can lead to various health issues, including cholangitis, cholelithiasis, cirrhosis, and even hepatobiliary carcinoma (Qian et al., 2016; Tang et al., 2016b). Currently, it is estimated that approximately 15-20 million people worldwide are affected by clonorchiasis, with the highest prevalence in China, South Korea, and northern Vietnam (Na et al., 2020). According to the World Health Organization (WHO), the global disease burden of clonorchiasis in 2010 was about 522,863 disability-adjusted life years (DALYs) (Chai et al., 2022). Zhao et al. calculated that the disease burden caused by clonorchiasis in China in 2016 was estimated to be 489,174.04 DALYs (Zhao and Lai, 2021). Additionally, *C. sinensis* has been classified as a Group I carcinogen by the WHO, and it is estimated that around 5,000 cases of cholangiocarcinoma (CCA) occur annually due to *C. sinensis* infection (Bouvard et al., 2009; Qian and Zhou, 2021a). However, the pathogenic mechanism of *C. sinensis* remains unclear, particularly regarding the molecular biological events that take place in the host liver.

Commonly used omics techniques include genomics, transcriptomics, proteomics, metabolomics, and epigenomics. Actually, network-based approaches for integrating multi-omics data are increasingly being applied to study disease pathogenesis, discover biomarkers for diagnosis, and predict therapeutic targets (Agamah et al., 2022; Chen et al., 2023). Currently, high-throughput omics technologies have been widely used in the research of parasite itself, parasite-host interaction, and prevention and control of parasitic diseases. For example, a combination of multi-omics has been used for the diagnosis and therapy of malaria (Tang et al., 2016b; Aggarwal et al., 2021; Zhou et al., 2021). Multi-omics techniques are applied to explore the interaction between *Opisthorchis viverrini* and the host, so as to control worm infection and prevent CCA (Prasopdee et al., 2019). The omics-based research on *Schistosoma* spp. has been well developed, and future omics-based new diagnostic tools for schistosomiasis have been proposed (Wang and Hu, 2014). With the rapid development of high-throughput technology, the complete genome, transcriptome, and secretome of *C. sinensis* has been extensively studied, providing a solid foundation for in-depth research on the biological characteristics of the parasite, as well as the screening and cloning of antigen candidates (Wang et al., 2011; Tang et al., 2016b; Young et al., 2021). Furthermore, the profound impact of early *C. sinensis* infection on the host has been well elucidated (Wu et al., 2023). However, little is known about the changes at the overall molecular level of the host caused by long-term parasitism of *C. sinensis*.

In the present study, we first investigated the effects of different stages of *C. sinensis* infection on mice, and evaluated the life quality and pathological conditions of mice at each stage. Then, we conducted a gene-protein-metabolism network analysis on the livers of mice at representative stage of *C. sinensis* infection by utilizing a combination of transcriptomics, proteomics, and metabolomics. Our results not only provide a more detailed interpretation of clonorchiasis, but also offer a comprehensive understanding of the molecular interaction between *C. sinensis* and its host.

## 2 Materials and methods

### 2.1 Ethics statement

All animal experiments were conducted in accordance with the guidelines for the Care and Use of Laboratory Animals in China, and approved by the ethical committee for animal research of Guangxi Medical University (approval no. 202308123).

### 2.2 Parasites

*C. sinensis* metacercaiae were obtained from naturally infected freshwater fish (*Pseudorasbora parva*), in Hengxian County, Guangxi Zhuang Autonomous Region, China. Living metacercariae were collected by routine digestion of fish in 0.8% pepsin solution with 0.2% HCl overnight at 37°C. Afterwards, filtered through 60-80 mesh sieve. Finally, the living metacercariae were isolated from clean sediment using an optical microscope and stored in PBS at 4°C (Xie et al., 2022).

### 2.3 Animal infection and sample collection

Female BALB/c mice, 6 weeks old, were purchased from the Hunan SJA Laboratory Animal Co., Ltd, and housed in a temperature-controlled room (25°C ± 2°C) with a 12:12 h light-dark cycle and fed standard chow. The mice were randomly divided into 8 groups and each group was composed of 5 animals (n=5). These groups were categorized based on four time points: 5 weeks (5 w), 10 weeks (10 w), 15 weeks (15 w), 20 weeks (20 w). Each time point included 2 groups: experimental group and control group. Each experimental mouse was orally infected with 60 living metacercariae and the control mice were administered the same volume of PBS (200 μl).

After 5 w, 10 w, 15 w and 20 w of intragastric administration, the corresponding mice were weighed and then sacrificed to collect samples of anticoagulant whole blood, serum and liver tissue. Meanwhile, the liver and spleen were separated and immediately weighed to calculate the liver/spleen index. The anticoagulated blood was used to complete the blood routine indexes test. The serum samples were subjected to detect cytokine levels by using ELISA. The left lobe liver tissues of mice were used for histopathological staining. Besides, the remaining left lobe liver tissues of mice in 5 w groups were promptly frozen in liquid nitrogen for RNA sequencing analysis, proteomics analysis and metabolomics analysis, respectively.

### 2.4 Determination of liver index and spleen index of mice

At each indicated time points, the body weight of each mouse was recorded before sacrificed and the liver/spleen weight was acquired immediately after the death of mice. The liver/spleen index was calculated by the following formula: liver/spleen index (mg/g) = liver/spleen wet weight (mg)/mouse body weight (g).

### 2.5 Histology staining

Mice were sacrificed at corresponding time points, then liver tissues of left middle lobe were fixed in 4% paraformaldehyde, embedded in paraffin and cut into 5 μm sections. All paraffin-embedded liver tissues were stained with hematoxylin and eosin (H&E) and Masson’s trichrome staining, respectively. Finally, the stained sections were observed and photographed under an optical microscope. The percentage of area positive for Masson’s trichrome staining was quantified using Image J software, and liver fibrosis was graded according to Ishak’s score (Ishak et al., 1995).

### 2.6 Biochemical indicators detection

The blood samples of mice from each group were collected and prepared for serum and anticoagulant blood. Serum hepatic enzyme activities were detected with an alanine aminotransferase (ALT) assay kit and an aspartate aminotransferase (AST) assay kit (Jiancheng, Nanjing, China), respectively. The levels of white blood cell (WBC), lymphocyte (LYM), granulocyte (GRAN), monocyte (MONO), red blood cell (RBC), hematocrit (HCT), hemoglobin (HGB), and mean corpuscular volume (MCV) were determined using a full-automatic biochemical analyzer (Rayto, Shenzhen, China) in Servicebio technology (Wuhan, China).

### 2.7 Enzyme-linked immunosorbent assay

To examine the changes in immune responses in each group, the levels of cytokines (IL-6, IL-1β, TNF-α, IL-4, and IL-10) in serum were quantified using ELISA Kits (Thermo, Massachusetts, USA) following the instructions provided. In addition, C-reactive protein (CRP) levels in serum samples were determined using a mouse CRP ELISA kit (MultiSciences, Hangzhou, China).

### 2.8 Transcriptomic analyses

To evaluate gene expression profiling, total RNA was extracted from the 5 w groups using MJzol Reagent (Invitrogen, Massachusetts, USA). Samples were sequenced on an Illumina Novaseq 6000 platform (San Diego, CA, USA) in Majorbio Bio-pharm Technology (Shanghai, China). After quality control, clean data (reads) were obtained to align to the reference genome. Differential expression analysis was performed using DESeq2. Hierarchical clustering was performed using Euclidean distance and average linkage method. The sample clustering was performed using the complete linkage method. Finally, Enrichment analyses were performed using the Goatools and KOBAS software, including differentially expressed genes (DEGs) cluster analysis, Gene Ontology (GO, http://geneontology.org/) term analysis and Kyoto Encyclopedia of Gene and Genomes (KEGG, http://www.genome.jp/kegg//) pathway enrichment analysis.

### 2.9 Proteomic analyses

Mouse liver tissues were collected at 5 w post infection for proteomic analyses. The proteins were detected by 4D Label Free Quantitative proteomics technology. The peptides were dissolved and analyzed by LC–MS/MS using EASY-nLC 1000 system (Thermo, Massachusetts, USA) with timsTOF Pro2 mass spectrometer (Bruker, Karlsruhe, Germany). The scan range of the MS/MS was set at 100 to 1700m/z. Data acquisition used the parallel accumulation serial fragmentation (PASEF) acquisition mode. The raw data were searched using MaxQuant software. All data were analyzed through the free online platform of majorbio cloud platform (cloud.majorbio.com).

Hierarchical clustering of the differentially expressed proteins (DEPs) was performed using Euclidean distance and average linkage method. DEPs were classified by GO annotation based on three categories: biological process (BP), cellular component (CC) and molecular function (MF). Pathways enrichment analysis of DEPs was conducted according to the KEGG pathway database. The STRING protein interaction database was used to analyze the protein–protein interaction (PPI) network.

### 2.10 LC-MS/MS based untargeted metabolomic analyses

Liver tissues were prepared from 5 w group for the detection of metabolites using untargeted Liquid Chromatography-Tandem Mass Spectrometry (LC-MS/MS). The following steps were conducted by Majorio Bio-Pharmm Technology Co., Ltd. (Shanghai, China). The reference method was as follows (Wang et al., 2019): briefly, after sample processing, the samples test was performed on the UHLC-Q Active HF-X system (Thermo, Massachusetts, USA). Samples were passed through HSS T3 column (Waters, Milford, USA) separated and then detected by mass spectrometry. Positive and negative ion scanning modes were adopted for sample mass spectrum signal acquisition. The parameters used were as follows: spray voltages 3.5 and -3.5 kV, respectively; scanning range 70-1050 m/z; normalized collision energy 20-40-60 V; resolution of the primary and secondary mass spectrometry 60000 and 7500, respectively. The DDA mode was utilized to collect data. Subsequently, the metabolic raw data were processed by Progenesis QI (Waters Corporation, Milford, USA) and a series of ways to obtain the data matrix for subsequent differentially expressed metabolites (DEMs) analysis, significant metabolites screening and KEGG pathway analysis, and DEMs used Euclidean distance and average linkage method for hierarchical clustering. Metabolic pathway analysis was carried out using the well-established mummichog algorithm.

### 2.11 Correlation analyses of transcriptomics, proteomics and metabolomics

Correlation analyses were conducted on the majorbio cloud platform to examine the relationships between DEGs and DEPs, DEGs and DEMs, as well as DEPs and DEMs using the transcriptomics, proteomics, and metabolomics datasets. The analysis encompassed Venn diagram analysis, cluster analysis, expression correlation analysis, and functional enrichment analysis.

### 2.12 Statistical analyses

All data were represented by mean ± standard deviation (SD). SPSS 23.0 software was used for Student’s *t*-test. *P* < 0.05 was considered to be statistically significant. For transcriptomic data, the filtering criteria for DEGs were set as *P*adjust < 0.05 and |FC| ≥ 2. Differential gene expression analysis was performed with DESeq2 software, and the Benjamini and Hochberg FDR (BH) method was used for multiple testing correction of *P*value. For proteomic analysis, Student’s *t*-test (two-tailed) was used with a *P*value < 0.05 and |FC| ≥ 2 as the filtering criteria. The gene set enrichment analysis was performed on hypergeometric algorithm. The significance of gene enrichment in GO analysis was determined based on a *P*adjust < 0.05, while *P*adjust < 0.5 was used for KEGG analysis. Similarly, the functional enrichment analysis of DEPs was based on the threshold of *P*value < 0.5. For metabolomic data, FC analysis and T test/nonparametric test were employed to analyze the difference between two groups of samples. DEMs for KEGG enrichment analysis based on *P* value or FDR < 0.05. The KernelDensity function was used to calculate correlations of DEGs and DEPs. DEGs and DEPs enrichment analysis was conducted using Diamond software, with a significance threshold of *P* ≤ 0.5. Correlation analysis of DEGs and DEMs was performed by calculating Pearson’s coefficient. *P* < 0.05 and the absolute value of correlation coefficient more than 0.8 were used as the cutoffs. The significantly enriched pathways of DEGs and DEMs were obtained using the hypergeometric distribution algorithm (*P* < 0.05). Pearson correlation coefficient and Fisher’s exact test were used for correlation analysis of DEPs and DEMs, as well as correlation analysis of pathway enrichment of proteomics and metabolomics, respectively.

## 3 Results

### 3.1 Effect of *C. sinensis* infection on liver and spleen indexes in mice

**Figure 1:**
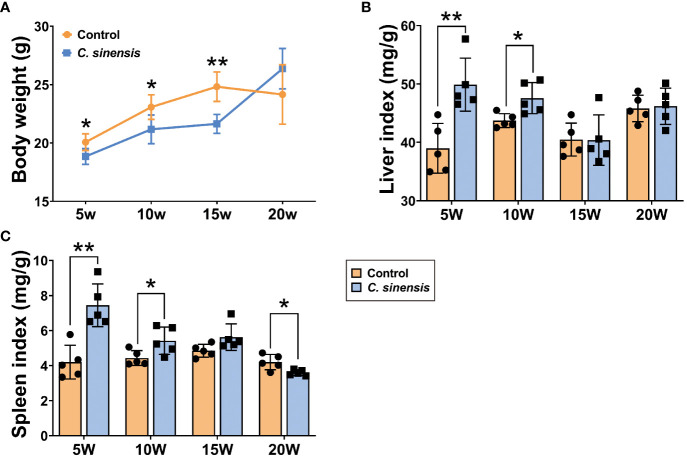
Liver index and spleen index of mice infected with *C. sinensis*. **(A)** Changes in body weight at different times of infection. **(B)** Liver index. **(C)** Spleen index. Data are shown as mean ± SD (n=5). *P < 0.05, **P < 0.01.

Compared with the control group, the body weights of mice in the infected group were significantly lower at all time points, except for week 20 (*P* < 0.05, **Figure 1A**). The liver and spleen indexes in the infected group showed significant increases at weeks 5 and 10 of infection (*P* < 0.05), with no statistical difference observed at week 15. Furthermore, the spleen indexes were significantly decreased at week 20 (*P* < 0.05, **Figures 1B, C**).

### 3.2 Gross and histopathological changes of mouse liver caused by *C. sinensis* infection

**Figure 2:**
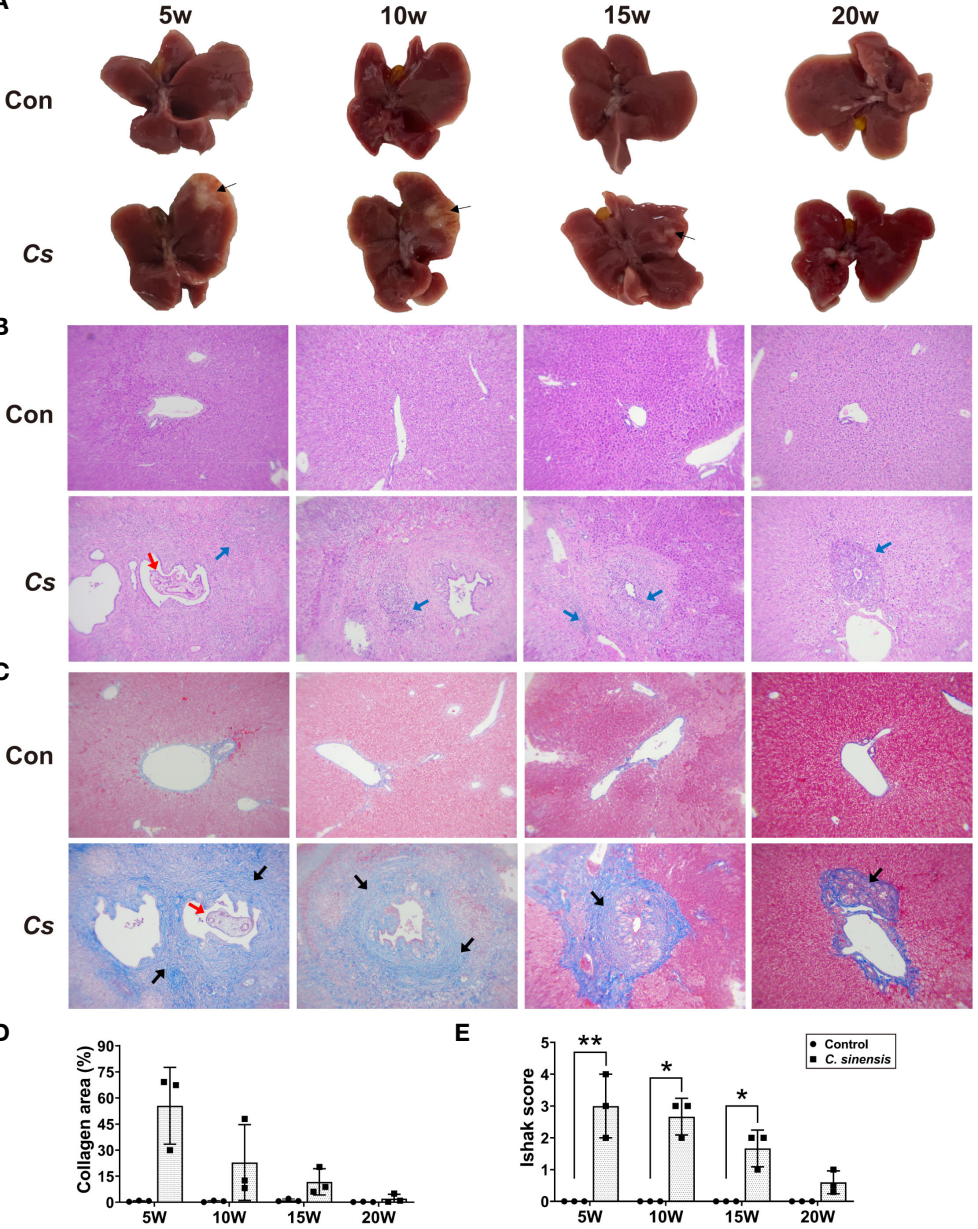
Gross and histopathological observation of liver in *C. sinensis*-infected mice. **(A)** Gross observation of the liver tissues of mice, thin black arrows indicate inflammatory foci. **(B)** H&E staining of mouse liver sections (×100). Red arrow indicates adult worm and blue arrows indicate inflammatory cell infiltration. **(C)** Masson trichrome staining of mouse liver sections (×100). Red arrow indicates adult worm, and heavy black arrows indicate collagen deposition. **(D)** Collagen area as percentage of tissue area. **(E)** Ishak fibrosis score. Data are shown as mean ± SD (n=3). *P < 0.05, **P < 0.01.

Obvious white foci with hard textures (thin black arrows) were observed in the livers of *C. sinensis* infected mice at both 5 and 10 weeks post-infection, especially in the left lobes. However, the symptoms gradually decreased starting from the 15th week, and no visible lesions were found after 20 weeks of infection (**Figure 2A**). Histological staining of mouse livers demonstrated that the most severe symptoms, including inflammatory cell infiltration (blue arrows), biliary duct hyperplasia, and collagen deposition (thick black arrows) occurred at 5 weeks of infection and gradually decreased afterwards (**Figures 2B, C**). In comparison to the control group, both the collagen-positive areas and Ishak scores of the infected mouse livers increased at each time point (**Figures 2D, E**). Importantly, the Ishak scores of Masson staining in the 5 w, 10 w and 15 w infected groups were significantly higher than those of the control groups (*P* < 0.05, **Figure 2E**).

### 3.3 Changes of biochemical enzymes, blood routine and cytokines in blood after *C. sinensis* infection

**Figure 3:**
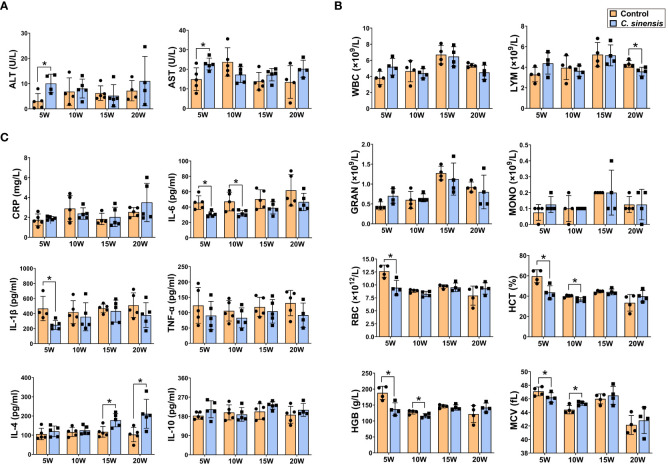
Levels of biochemical enzymes, blood routine indicators and cytokines in mouse blood. The mice in groups of 5 w, 10 w, 15 w and 20 w were sacrificed at corresponding time points to prepare serum or anticoagulated blood for determination of serum biochemical enzymes of ALT and AST **(A)**, blood routine indicators of WBC, LYM, GRAN, MONO, RBC, HCT, HGB, and MCV **(B)** and cytokine levels of CRP, IL-6, IL-1β, TNF-α, IL-4, and IL-10 **(C)**, respectively. Data are presented as mean ± SD (n=5). *P < 0.05.

Serological tests revealed significant increases in both ALT and AST at 5 weeks post infection (*P* < 0.05, **Figure 3A**). Blood routine data demonstrated an initial increase in the number of WBC, LYM, GRAN, and MONO at week 5 after infection, followed by a decline in these numbers. All indicators of RBC, HCT, HGB, and MCV were significantly decreased at week 5 (*P* < 0.05, **Figure 3B**), but showed varying degrees of recovery afterwards, especially at 20 weeks of infection. ELISA results showed decreased levels of inflammatory cytokines IL-6, IL-1β, and TNF-α at each infection time point. Specifically, the levels of IL-6 were significantly lower at weeks 5 and 10, and the levels of IL-1β were significantly lower at week 5 (*P* < 0.05, **Figure 3C**). Additionally, the levels of anti-inflammatory cytokines IL-4 and IL-10 tended to increase at all time points, with significant increases in IL-4 at the 15 and 20 weeks of infection (*P* < 0.05, **Figure 3C**).

### 3.4 DEGs in mouse liver induced by *C. sinensis* infection

**Figure 4:**
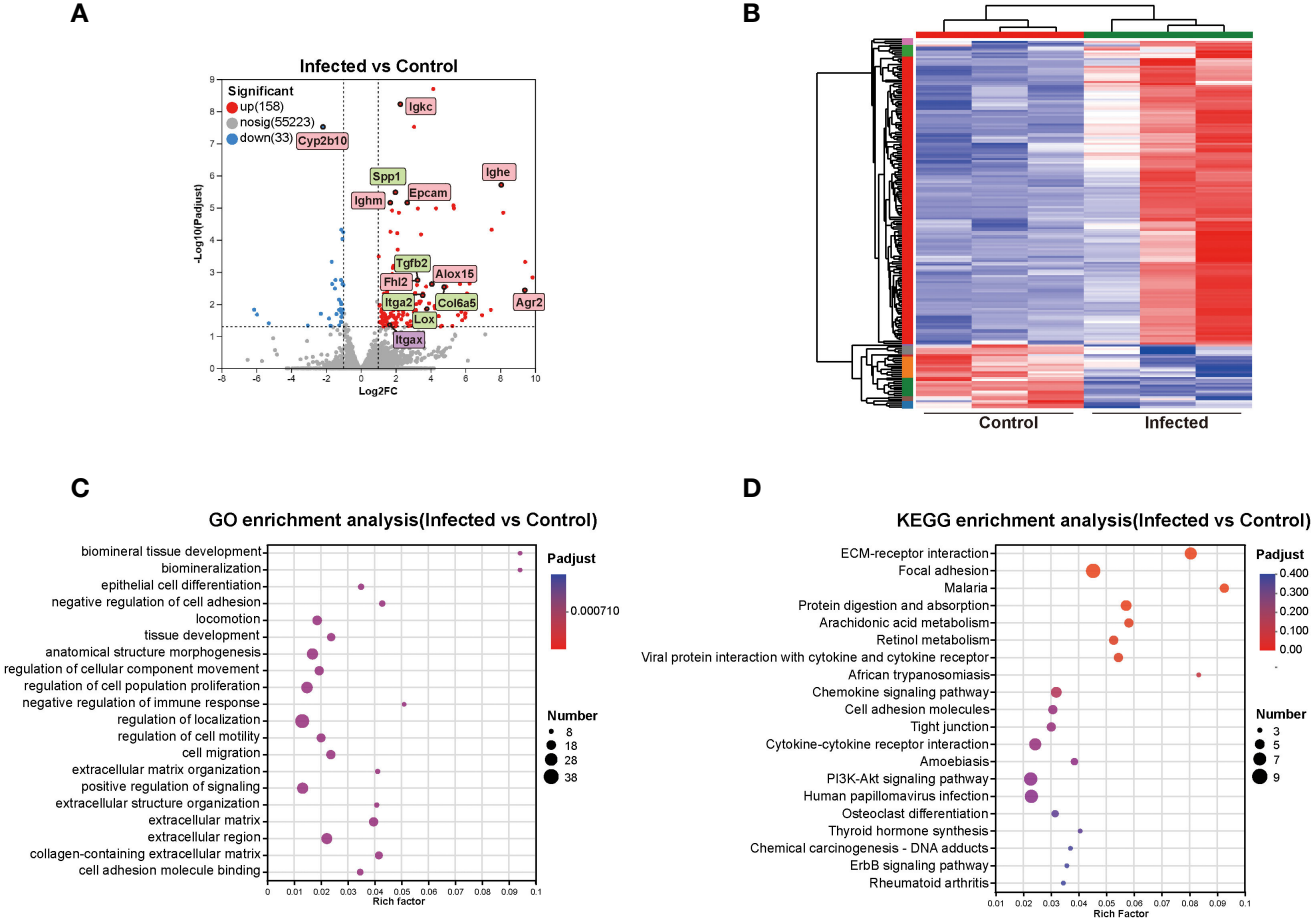
Cluster and enrichment analysis of mouse liver DEGs at 5 weeks post *C. sinensis* infection. **(A)** Volcano diagram of DEGs analysis. **(B)** Heatmap of DEGs between infected and control group. **(C)** GO enrichment analysis of DEGs. **(D)** KEGG pathway enrichment analysis of DEGs. The top 20 items of GO and KEGG enrichment analyses are shown.

To elucidate the mechanism of the effects of *C. sinensis* on mouse liver, transcriptomics profiling of 5 w groups were performed. A total of 191 DEGs were detected, with 158 up-regulated genes and 33 down-regulated genes (FC ≥ 2, *P* < 0.05, **Figure 4A**). DEGs significantly involved in GO and KEGG enrichment (green rectangles) of transcriptomics, as well as common molecules significantly involved in GO and KEGG enrichment in both transcriptomics and proteomics (pink rectangles), were marked on volcano diagram (**Figure 4A**). The cluster heatmap displayed all DEGs of the infected group compared with the control group (**Figure 4B**).

The top 20 enrichment results in each category of GO analysis and in KEGG pathways were displayed in **Figures 4C, D**. The predominant enriched GO terms of DEGs included biomineral tissue development, collagen-containing extracellular matrix, extracellular matrix organization, negative regulation of immune response, negative regulation of cell adhesion, cell adhesion molecule binding, and epithelial cell differentiation (**Figure 4C**). The primary enriched KEGG pathways included ECM-receptor interaction, focal adhesion, protein digestion and absorption, arachidonic acid metabolism, retinol metabolism, chemokine signaling pathway, and cell adhesion molecules (CAMs) (**Figure 4D**). Genes such as Alox15, Lox, Tgfb2, Epcam, Col6a5, Col4a5, Olfm4, Agr2, Itga2, Cd34, Ccl11, Itgax, Spp1, Cyp2b10, Fhl2, Mmp7, and Esm1were primarily participated in aforementioned GO items and KEGG pathways (**Figure 4**).

### 3.5 Annotation and functional enrichment of DEPs in the liver of *C. sinensis* infected mice

**Figure 5:**
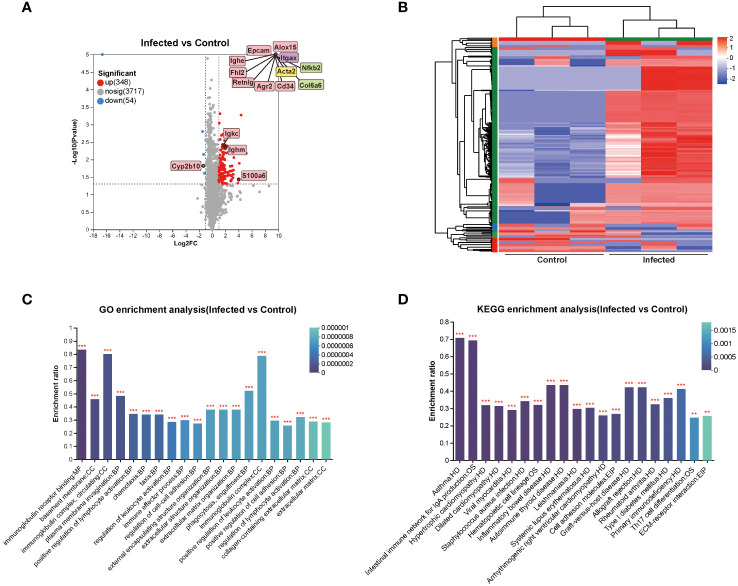
Cluster and enrichment analysis of mouse liver DEPs at 5 weeks of *C. sinensis* infection. **(A)** Volcano diagram of DEPs analysis. **(B)** Cluster heatmap of the DEPs. **(C)** Enriched GO terms of the identified DEPs. **(D)** Enriched KEGG pathways of the DEPs. The top 20 items of GO and KEGG enrichment analyses are shown. The color gradient indicated the significance of enrichment, and the darker the color indicated the higher the enrichment degree (***P < 0.001, **P < 0.01).

To gain further insights into the DEPs between the mouse livers of 5 w infected group and 5 w control group, proteomics analysis was conducted. The volcano plot revealed 348 up-regulated DEPs and 54 down-regulated DEPs, with an absolute value of FC ≥ 2 and *P* < 0.05. The DEPs that significantly participated in GO and KEGG enrichment of proteomics (green rectangles) and the common molecules significantly involved in GO and KEGG enrichment of both the transcriptomics and proteomics (pink rectangles) were marked on volcano diagram, respectively (**Figure 5A**). For PPI network analysis, nodes indicated proteins, and dotted lines indicated the interaction between nodes. The central protein, Acta2 (yellow rectangle), in the PPI network and the central molecule, Igtax (purple rectangle), in the transcriptional protein-associated PPI network were also indicated on the volcano map (**Figures 4A**, **5A**). The interaction among DEPs was demonstrated in **Supplement Figure 1**. The cluster heatmap presented all the DEPs between 5 w infected group and 5 w control group (**Figure 5B**).

The results of GO and KEGG enrichment analysis of DEPs were represented as bar graph (**Figures 5C, D**). The primarily enriched GO terms included immunoglobulin receptor binding, basement membrane, immunoglobulin complex, circulating, positive regulation of lymphocyte activation, chemotaxis, regulation of cell-cell adhesion, extracellular matrix organization, and collagen-containing extracellular matrix. The main KEGG pathways involved were CAMs, ECM-receptor interaction, asthma, intestinal immune network for IgA production, Th17 cell differentiation, inflammatory bowel disease, autoimmune thyroid disease, rheumatoid arthritis, and hematopoietic cell lineage. The DEPs that participated in the aforementioned GO and KEGG enrichments were mainly Alox15, Epcam, Retnlg, Acta2, Cd34, Itgax, Ighe, Ighm, Igkc, Col6a6, Col15a1, Nfkb2, S100a6, Alox5, Vcam1, and Cd44 (**Figure 5**).

### 3.6 Correlation analysis of transcriptomics and proteomics after *C. sinensis* infection

**Figure 6:**
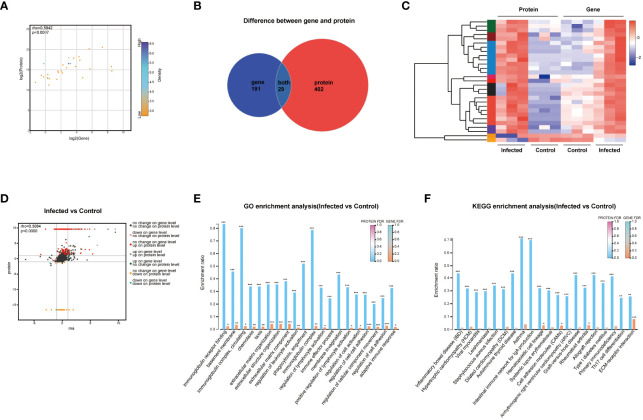
Correlation analysis of transcriptomics and proteomics. **(A)** Scatter plot of correlation between transcriptomics and proteomics. **(B)** Venn diagram of transcriptomics and proteomics. **(C)** Heatmap of transcriptomics-proteomics correlation analysis. **(D)** Nine-quadrant diagram of transcriptomics and proteomics. **(E)** Functional categories enrichment analysis at both transcriptomics and proteomics levels. **(F)** Pathways enrichment analysis at both transcriptomics and proteomics levels. FDR < 0.001, FDR < 0.01 and FDR < 0.05 were marked ***, ** and * respectively.

The correlation analysis results demonstrated a significant correlation between the transcriptomics and proteomics (rho=0.5942, *P* < 0.0007, **Figure 6A**). The Venn analysis and cluster heatmap analysis revealed that there were 29 DEGs/DEPs shared between the transcriptomics and proteomics datasets, exhibiting consistent changes in expression patterns (**Figures 6B,C**). The detailed information of these 29 shared molecules was displayed in **Supplement Table 1**. Additionally, the nine-quadrant diagram showed the changes of up-/down-regulated genes/proteins (**Figure 6D**). The central protein of correlation analysis was Itgax (**Supplement Figure 2**).

GO enrichment analysis showed the six most significantly enriched GO terms at both transcriptomics and proteomics levels, including extracellular matrix organization, extracellular structure organization, extracellular matrix component, regulation of cell-cell adhesion, regulation of cellular component movement, and regulation of cell adhesion (*P* < 0.001). In addition, the GO terms of immunoglobulin receptor binding, basement membrane, immunoglobulin complex, circulating, chemotaxis, and regulation of leukocyte activation also showed high enrichment (**Figure 6E**). KEGG analysis revealed that pathways of ECM-receptor interaction, CAMs, hematopoietic cell lineage, and rheumatoid arthritis were highly significantly co-enriched between transcriptomics and proteomics (**Figure 6F**).

### 3.7 DEMs and KEGG functional enrichment analysis of the liver after *C. sinensis* infection

**Figure 7:**
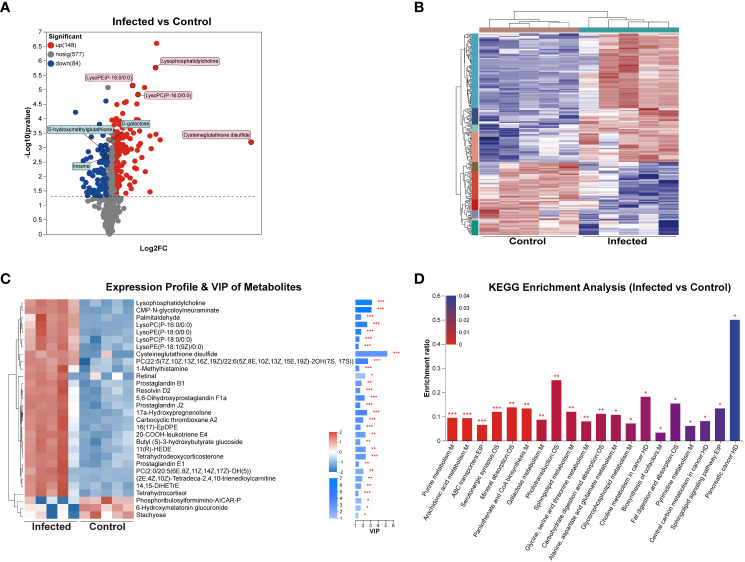
DEMs analysis of mouse liver at 5 weeks post *C. sinensis* infection. **(A)** Volcano diagram of DEMs. **(B)** Cluster analysis of all DEMs. **(C)** Variable importance in projection (VIP) scores of DEMs. **(D)** KEGG enrichment analysis of the DEMs between infected and control group. *P* value or FDR < 0.001, *P* value or FDR < 0.01 and *P* value or FDR < 0.05 were marked ***, **, *, respectively.

To investigate the effects of *C. sinensis* infection on liver metabolites, liver samples of 5w groups subjected to untargeted LC-MS/MS. The RSD results of QC samples confirmed the reliability of the data obtained in the experiment (**Supplement Figure 3**). The results of PCA, PLS-DA, and OPLS-DA models demonstrated excellent experimental repeatability and revealed differences in metabolites between the two groups (**Supplement Figure 4**). In total, 809 metabolites were identified, with 473 in positive ion mode and 336 in negative ion mode. A total of 232 DEMs were detected, with 148 up-regulated and 84 down-regulated, and representative up-regulated/down-regulated metabolites were labeled in pink rectangles and blue rectangles, respectively (**Figure 7A**). The cluster analysis of DEMs was presented in **Figure 7B**. VIP analysis identified 30 DEMs that significantly contributed to the grouping (VIP > 2, *P* < 0.05). The top 5 metabolites were cysteine-glutathione disulfide, lysophosphatidylcholine (LysoPC), CMP-N-glycoloylneuraminate, PC(22:5(7Z,10Z,Z,16Z,19Z)/22:6(5Z,8E,10Z,13Z,15E,19Z)-2OH(7S, 17S)), and LysoPC (P-16:0/0:0) (**Figure 7C**).

KEGG enrichment analysis confirmed that *C. sinensis* infection significantly affected metabolic pathways including purine metabolism, arachidonic acid metabolism, ABC transporters, mineral absorption, pantothenate and CoA biosynthesis, galactose metabolism, sphingolipid metabolism, and glycine, serine, and threonine metabolism. The DEMs involved in these KEGG enrichment pathways included inosine, 14,15-DiHETrE, L-glycine, L-serine, D-galactose, Prostaglandin J2 and L-glutamine (**Figure 7D**).

### 3.8 Correlation analysis of transcriptomics and metabolomics, and proteomics and metabolomics after *C. sinensis* infection

**Figure 8:**
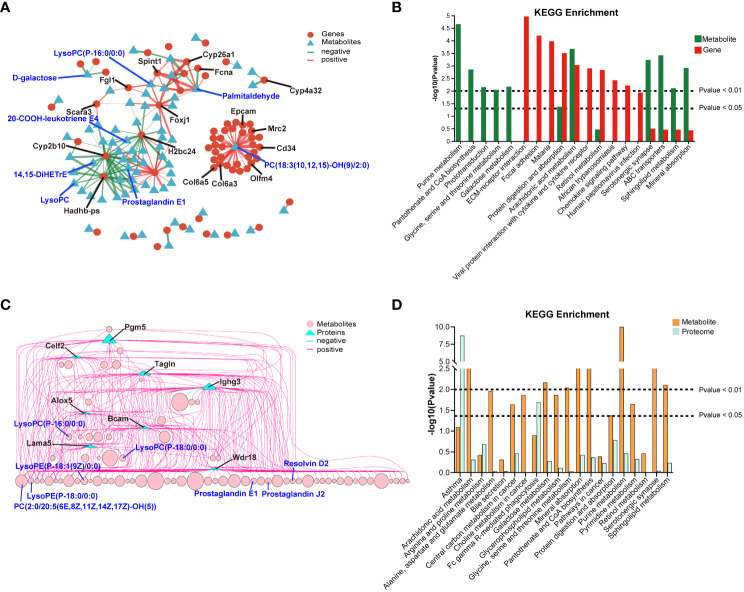
Correlation analysis of between transcriptomics and metabolomics, and proteomics and metabolomics. **(A)** Network diagram of the correlation between DEGs and DEMs. **(B)** 20 co-enriched pathways of transcriptomics and metabolomics. **(C)** Network diagram of the correlation between DEPs and DEMs. **(D)** 20 co-enriched pathways of proteomics and metabolomics.

The correlation network between DEGs and DEMs was shown in **Figure 8A**, and detailed data were presented in **Supplement Table 2**. The correlation analysis revealed that several pathways, including arachidonic acid metabolism, protein digestion and absorption, ABC transporters, sphingolipid metabolism, mineral absorption, and retinol metabolism, were co-enriched in both the transcriptomics and metabolomics (*P* < 0.05, **Figure 8B**). The correlation network between DEPs and DEMs was presented in **Figure 8C**, and detailed data were shown in **Supplement Table 3**. The correlation analysis of the proteomics and metabolomics demonstrated that the co-enriched pathways were arachidonic acid metabolism, purine metabolism, sphingolipid metabolism, mineral absorption, central carbon metabolism in cancer, and Fc gamma R-mediated phagocytosis (**Figure 8D**).

## 4 Discussion

According to reports, it takes approximately one month for *C. sinensis* juveniles to develop into adults within the host’s hepatobiliary ducts (Qian et al., 2016; Koda et al., 2022). We constructed mouse models infected with *C. sinensis* from 5 to 20 weeks. Our results revealed that adult *C. sinensis* not only resulted in significant weight loss in mice, but also caused hepatosplenomegaly, leading to severe pathological damage to the liver. However, these symptoms gradually improved after 15 weeks of infection. Serological testing demonstrated significant increases in the levels of ALT and AST at 5 weeks of infection, indicating severe liver injury (Sookoian and Pirola, 2015). Blood routine data showed significant decreases in RBC, HCT, HGB, and MCV indices at 5 weeks post-infection, suggesting the occurrence of anemia (He et al., 2022). Cytokine assay confirmed that as the infection time prolonged, the levels of inflammatory cytokines (especially IL-6 and: IL-1β) decreased, while anti-inflammatory cytokines (especially IL-4) increased. These results were consistent with previous studies by Wang et al. and Kong et al., which demonstrated that Th2-type immune responses are primarily triggered during the adult stage of *C. sinensis*, aiding in the control of excessive inflammation and promoting tissue repair (Kong et al., 2020; Wang et al., 2021; Koda et al., 2022). These results indicated that the parasitism of adult *C. sinensis* leads to severe liver damage, anemia, and systemic diseases in the host, particularly at 5 weeks post-infection. The symptoms gradually improved after 15 weeks, which could be attributed to factors such as the sustained increase in Th2 immune responses in the host and the death of parasites at the later stages of infection. It has been documented that adult *C. sinensis* can be chronically parasitized in humans for up to 20-25 years (Tang et al., 2016b). We analyzed the reasons for the short survival time of *C. sinensis* in mice. On one hand, mice have strong resistance to the parasite’s attack, and on the other hand, the narrow biliary space of mice and the increasingly harsh living environment as the infection progresses (such as anemia leading to nutrient deficiency, etc.) are not conducive to the long-term survival of the parasite. However, further in-depth research is required to support this speculation. Consequently, we selected liver tissues infected for 5 weeks for further multi-omics analysis.

Repeated or persistent chronic liver injury leads to inflammation and fibrosis of the liver. Liver fibrosis is characterized by excessive deposition of ECM proteins, resulting in the formation of fibrous scarring. Myofibroblasts (MFBs) are the primary source of ECM in fibrotic liver, with activated hepatic stellate cells (HSCs) and activated portal fibroblasts account for 90% of collagen-producing cells (Kisseleva and Brenner, 2021; Zhang et al., 2022). The expression of various molecular factors, such as CAMs and chemokines, is closely related to events during fibrous scar formation, including HSC proliferation, activation, migration, and leukocyte recruitment (Hintermann and Christen, 2019; Li, 2023). Our correlation analysis revealed a significant positive correlation between transcriptomics and proteomics data. Biomics were significantly enriched in GO items (ECM organization and ECM component) and KEGG pathways (ECM-receptor interaction and CAMs) that are closely associated with liver fibrosis. Moreover, multiple biological processes involved in the regulation or promotion of liver fibrosis progression, such as immunoglobulin complex, chemotaxis, regulation of leukocyte activation, and regulation of cell-cell adhesion, were significantly enriched at both the transcriptional and protein levels. Interestingly, correlation analysis of KEGG demonstrated significant enrichment of multiple autoimmune disease-related pathways such as rheumatoid arthritis, asthma, inflammatory bowel disease, and Th17 cell differentiation. Th17 cell differentiation pathway has been closely related to hepatitis, fibrosis, and autoimmune diseases (Beringer and Miossec, 2018; Kartasheva-Ebertz et al., 2022). Therefore, these findings explain the “hygiene hypothesis” to some extent, but further in-depth research is necessary (Bach, 2017; Caraballo, 2018).

Correlation analysis of multi-omics revealed that pathways of arachidonic acid metabolism, sphingolipid metabolism, protein digestion and absorption, mineral absorption, and bile secretion were enriched at the gene, protein and metabolic levels after *C. sinensis* infection. It has been well documented that arachidonic acid metabolism and sphingolipid metabolism are both hallmarks of liver inflammation and key drivers of fibrosis (Ishay et al., 2020; Liu et al., 2022b). Furthermore, single metabolomic analysis demonstrated significant effects of *C. sinensis* infection on liver nucleotide metabolism (purine and pyrimidine), galactose metabolism, amino acid metabolism (glycine, serine, threonine, alanine, and aspartate and glutamate), ABC transporters, pantothenate and CoA biosynthesis, and glycerophospholipid metabolism. Purine and pyrimidine metabolites have been reported as metabolic messengers of the gut microbiota associated with liver cirrhosis (Xiong et al., 2022). Galactose is a crucial carbohydrate for cellular metabolism, contributing to energy production and tissue storage (Conte et al., 2021). Research reports have confirmed the close relationship between abnormal galactose metabolism and liver fibrosis and hepatocellular carcinoma (HCC) (Leonardi et al., 2007; Tang et al., 2016a). Pantothenate is a key precursor for CoA biosynthesis, an essential cofactor in numerous metabolic reactions, including phospholipid synthesis and fatty acid synthesis and degradation (Leonardi et al., 2007). Additionally, it has been well documented that ABC transporters expressed at the canalicular membrane of hepatocytes mediate the secretion of bile constituents and play a critical role in bile formation and cholestasis (Cuperus et al., 2014; Ben Saad et al., 2021). Moreover, metabolic pathways related to choline and central carbon metabolism in cancer were also enriched. These findings indicated that *C. sinensis* invasion significantly alters the metabolism of lipids, carbohydrates, amino acids, and nucleotides in the liver, as well as mineral absorption, bile secretion, further promoting the development of liver diseases.

In total, there were 29 molecules related to liver fibrosis, inflammation, immunity, and tumorigenesis showed consistent expression at both the gene and protein levels. Biomics results revealed significant upregulation of Cd34, Epcam, and S100a6, all of which are biomarkers for HSCs or MFBs activation during liver fibrogenesis (Terris and Perret, 2010; Krenkel et al., 2019; Wang et al., 2022). After *C. sinensis* infection, the local adhesion molecule Fhl2 was upregulated, promoting the expression of ECM proteins (Kostallari et al., 2022). The hepatic inflammatory marker Itgax and pro-inflammatory molecule Retnlg were also significantly upregulated (Valcarcel et al., 2006). Additionally, abundant molecules related to immunoglobulins, including the constant and variable regions genes of Igkc and Igkv3-7 (Pinheiro et al., 2016), dimer and pentamer assembly gene of Jchain (Pan et al., 2021), the anti-infective antibody gene of Ighg1 (Napodano et al., 2020), mediating innate and adaptive immunity antibody gene of Ighm (Weill and Reynaud, 2020), and the parasite-specific antibody gene of Ighe (Fitzsimmons et al., 2014), were significantly stimulated by *C. sinensis*. Molecules related to HCC formation, growth, metastasis, and immune evasion such as Agr2, Alox15, Mrc2, and Fgl1, were significantly upregulated (Hong et al., 2020; Qian et al., 2021b; Liu et al., 2022a). However, the expression of Cyp2b10 was significantly reduced, indicating hepatic metabolic disorders and abnormal detoxification function caused by *C. sinensis* infection (Chen et al., 2021). Additionally, at the single-omics level, a myriad of molecules associated with HSC activation and fibrogenesis were upregulated, including genes such as Lox, Tgfb2, Spp1, Col4a5, Col5a2, Col6a3, Col6a5, and Mmp7, and proteins such as Col15a1, Col6a6, Vcam1, and Nfkb2 (Kisseleva and Brenner, 2021; Sun et al., 2021; Unamuno et al., 2021; Guo et al., 2022; Dai et al., 2023).

Correlation analysis between transcriptomics and metabolomics, as well as proteomics and metabolomics, revealed a complex gene-protein-metabolism regulatory network triggered by *C. sinensis* infection. The analysis showed that 32 genes were positively correlated with PC(18:3(10,12,15)-OH(9)/2:0), especially genes of Col6a3, Col6a5, Olfm4, Mrc2, Cd34, and Epcam (corr > 0.99). Additionally, genes of Fgl1, Foxj1, and Spint1 were positively correlated with LysoPC(P-16:0/0:0). Phosphatidylcholine (PC) is a widely distributed phospholipid in eukaryotic cell membranes. Under oxidative conditions, it pathologically breaks down into LysoPC, which serves as the biomarker of several liver diseases (Qin and Hu, 2009; Hu et al., 2023). LysoPC(P-16:0/0:0), a potent inflammatory mediator, is involved in immunoregulation of multiple biological processes and has been found to be significantly overexpressed in injured liver (Hu et al., 2023). The downregulated Cyp2b10 gene showed a negative correlation with 26 metabolites, including LysoPC, 20-COOH-leukotriene E4, 14,15-DiHETrE, and prostaglandin E1. Reports have documented that leukotriene E4 can drive hepatocyte ER stress, and DiHETrE can mediate inflammation-related oxidative stress to promote the progression of liver cirrhosis and HCC (Lu et al., 2018; Anton et al., 2023). While, exogenous administration of prostaglandin E1 has been reported to improve liver fibrosis (Jin et al., 2015). In the analysis of protein-metabolite correlation, proteins of Bcam, Alox5, and Lama5 were significantly correlated with 42, 24, and 23 metabolites, respectively, such as LysoPC(P-16:0/0:0) and PC(2:0/20:5(6E,8Z,11Z,14Z,17Z)-OH(5)). Alox5, Lama5, and Bacm are functional molecules that regulate the progression of fibrosis or HCC (Kikkawa et al., 2008; Shajari et al., 2015; Batzdorf et al., 2022). Ighg3, an important component of immunoglobulin complexes, was significantly associated with 61 metabolites, including prostaglandin E1, prostaglandin J2, and resolvin D2, which can alleviate liver inflammation and fibrosis (Jin et al., 2015; Fitzgerald et al., 2023; Han et al., 2023). Therefore, multi-omics analysis confirmed that *C. sinensis* primarily caused severe inflammation and fibrotic reactions in the host liver. However, negative feedback regulations were also activated to suppress excessive inflammatory damage.

High-throughput multi-omics technologies offer new perspectives for exploring parasite-host interaction and parasitic diseases (Cantacessi et al., 2012). Currently, there are few reports on use of single omics approaches to study the gene or metabolite profiling of host samples after *C. sinensis* infection. Han et al. discovered that DEGs in the mouse livers infected for 4 weeks were mainly enriched in the FOXO, Wnt, and AMPK pathways (Han et al., 2022). Although these pathways were also identified in our KEGG enrichment analysis, but they did not rank in the top 20. The pathways that ranked higher in our study were ECM-receptor interaction, focal adhesion, and arachidonic acid metabolism, suggesting a transition from oxidative damage to progressing liver fibrosis from 4 to 5 weeks post-infection. Han et al. also investigated the serum metabolic profiling of rats infected with *C. sinensis*. Although their samples were different from ours, significant DEMs such as PCs and pathways such as alanine, aspartate and glutamate metabolism, glycerophospholipid metabolism, and pyrimidine metabolism were found to be consistent (Han et al., 2023). However, the biological information obtained from a single omics approach is limited. A more comprehensive and in-depth exploration of the key molecular events and related genes involved in liver injury after *C. sinensis* infection could be achieved by utilizing multi-omics analysis.

## 5 Conclusion

This study found that from 5 to 20 weeks, the symptoms of mice infected with *C. sinensis* were most severe in the 5th week, manifested as significant weight loss, hepatosplenomegaly, and severe hepatobiliary lesions. In multi-omics analysis of the liver infected for 5 weeks, transcriptomics and proteomics analyses jointly revealed a significant enrichment of fibrosis-related pathways such as ECM-receptor interaction and CAMs. Additionally, metabolomics analysis showed that the top 3 metabolic pathways were purine metabolism, arachidonic acid metabolism, and ABC transporters. Overall, *C. sinensis* induces a complex gene-protein-metabolism regulatory network in the host liver. This study provides a comprehensive view of the pathogenic mechanism of *C. sinensis* and offers new insights for the intervention of clonorchiasis.

## Data availability statement

The datasets presented in this study can be found in online repositories. The names of the repository/repositories and accession number(s) can be found in the article/**Supplementary Material**.

## Ethics statement

The animal study was approved by the ethical committee for animal research of Guangxi Medical University (approval no. 202308123). The study was conducted in accordance with the local legislation and institutional requirements.

## Author contributions

TZ: Conceptualization, Data curation, Formal Analysis, Methodology, Visualization, Writing – original draft. YW: Conceptualization, Data curation, Formal Analysis, Methodology, Visualization, Writing – original draft. XD: Conceptualization, Data curation, Formal Analysis, Methodology, Visualization, Writing – original draft. QL: Investigation, Methodology, Validation, Writing – original draft. YC: Investigation, Methodology, Validation, Writing – original draft. JL: Methodology, Validation, Writing – original draft. JW: Methodology, Validation, Writing – original draft. SL: Methodology, Validation, Writing – original draft. ZW: Project administration, Writing – review & editing, Resources, Supervision. DL: Project administration, Resources, Supervision, Writing – review & editing. ZT: Funding acquisition, Project administration, Resources, Supervision, Writing – original draft, Writing – review & editing.
