# Supplementary material for: Multi-omics approaches reveal the molecular mechanisms underlying the interaction between Clonorchis sinensis and mouse liver
Source: Front Cell Infect Microbiol. 2023 Nov 24;13:1286977. doi: 10.3389/fcimb.2023.1286977 (PMC10710275; doi:10.3389/fcimb.2023.1286977)
Supplement: Supplementary file 1 [file DataSheet_1.pdf]

Supplementary Material

1 Supplementary Figures and Tables

1.1 Supplementary Figures

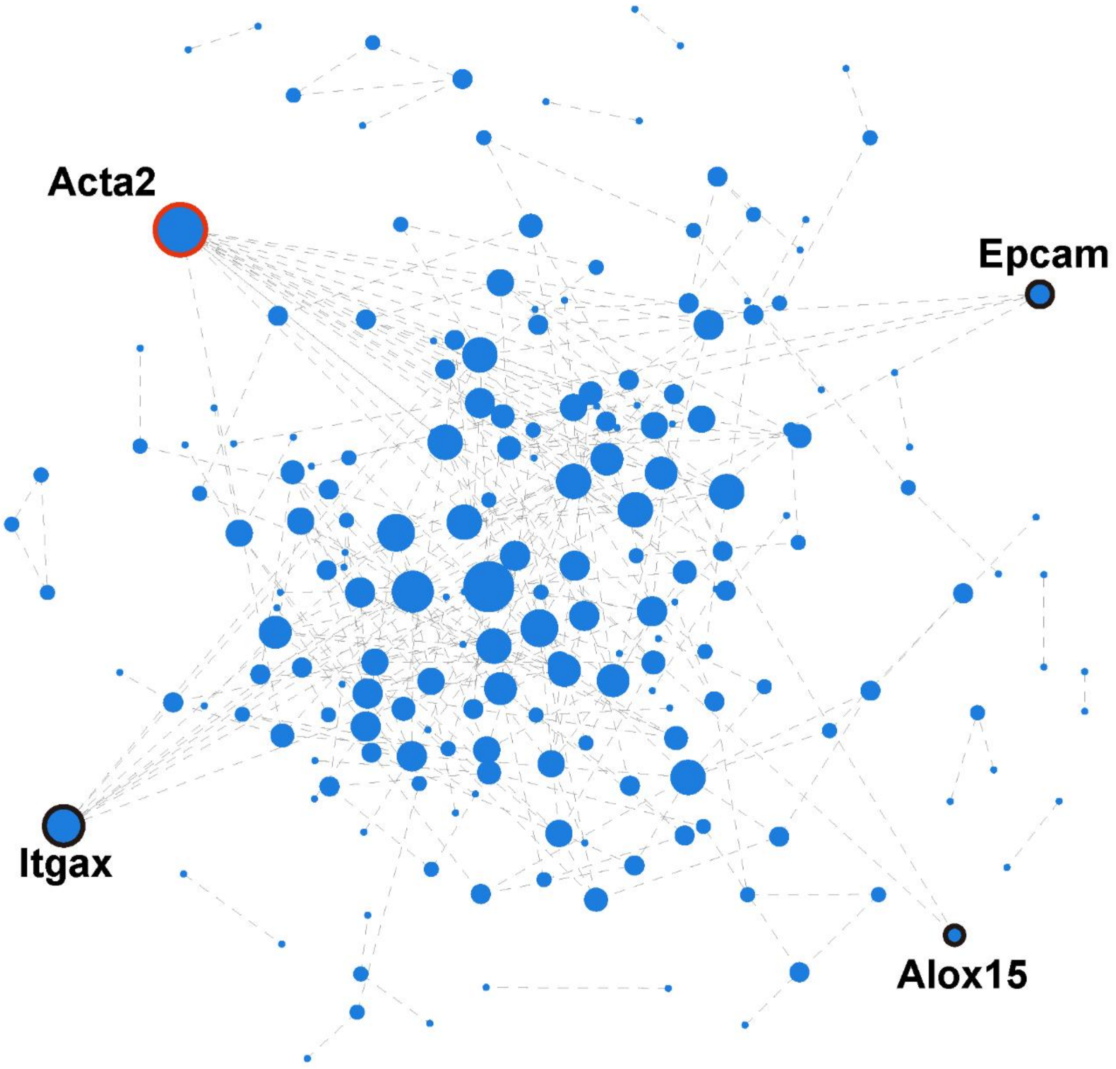

Figure S1. PPI network analysis of the DEPs between infected and control group at week 5. Red circle indicates the central protein.

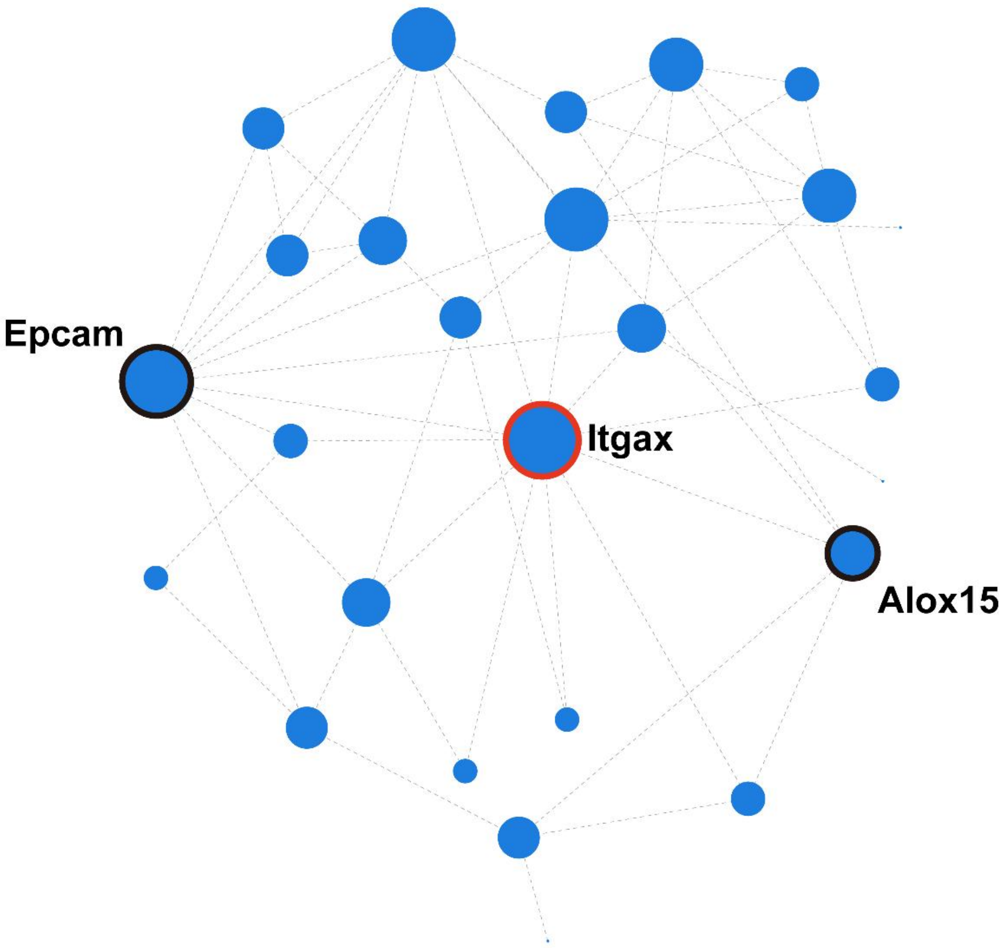

Figure S2. PPI network analysis of the common molecules shared by transcriptomics and proteomics. Red circle indicates the central molecule.

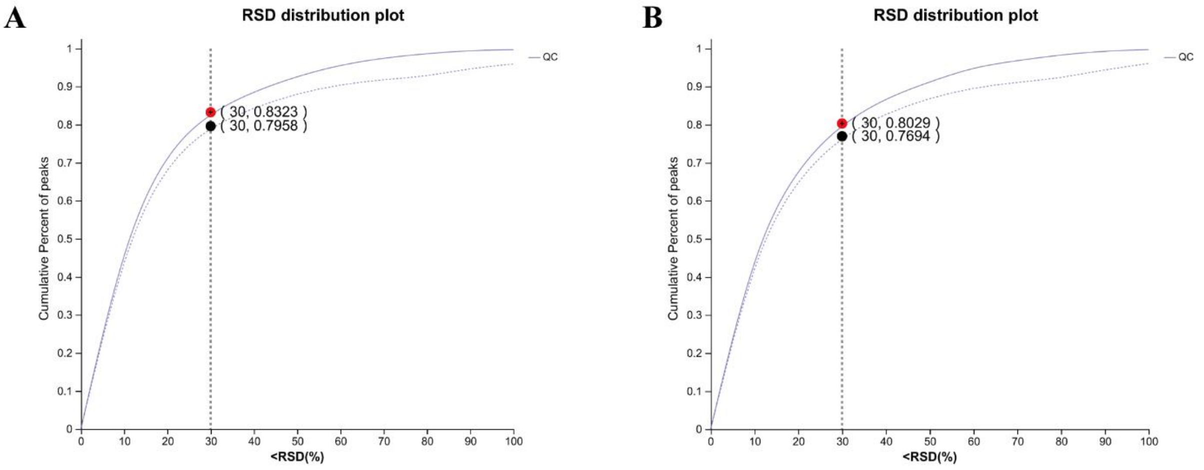

Figure S3. RSD evaluation of quality control (QC) samples for 5 weeks of infection.

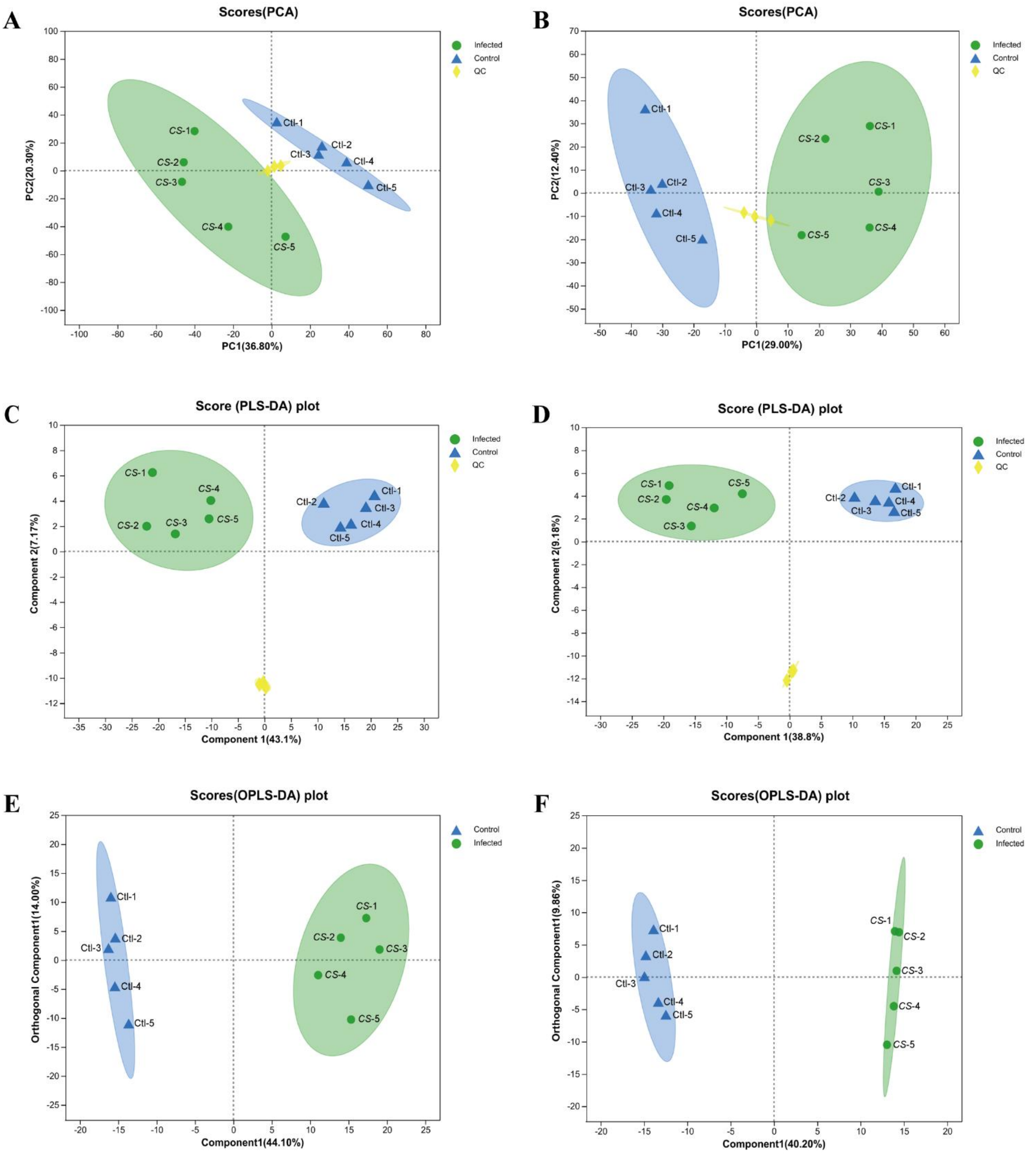

Figure S4. Differences in the composition of metabolites between infected and control group at week 5. Score diagrams of principal component analysis (PCA) **(A-B)**, partial least squares discrimination analysis (PLS-DA) **(C-D)**, and orthogonal partial least squares-discriminant analysis (OPLS-DA) **(E-F)** models in positive and negative mode.

1.2 Supplementary Tables

Table 1. DEGs and DEPs correlation analysis hierarchical clustering heatmap data.

Table 2. Correlation network list of DEGs and DEMs.

Table 3. Correlation network list of DEPs and DEMs.

2 Date availability statement

The raw RNA-seq data has been deposited to NCBI database under the accession number PRJNA1002127. The mass spectrometry proteomics data have been deposited to the Proteome Xchange Consortium via the iProX partner repository with the dataset identifier IPX0006894000.
